# Discharge readiness of elderly patients with osteoporotic vertebral compression fractures: a latent profile analysis

**DOI:** 10.3389/fmed.2026.1783513

**Published:** 2026-06-30

**Authors:** Ruyu Liao, Lili Chen, Shanhong Liu, Yuanyuan Liu, Yue Yang, Lan Chen

**Affiliations:** Department of Orthopaedics, The Affiliated Hospital of Southwest Jiaotong University, The Third People's Hospital of Chengdu, Chengdu, Sichuan, China

**Keywords:** discharge planning, discharge readiness, elderly, osteoporotic fracture, transitional care

## Abstract

**Objective:**

To Identify potential categories of discharge readiness among elderly patients with osteoporotic vertebral compression fractures, and analyze differences across categories.

**Methods:**

Between January 2025 and April 2025, a total of 356 elderly OVCF patients were included. The following data were collected: general information, the Readiness for Hospital Discharge Scale (RHDS), and the Quality of Discharge Teaching Scale (QDTS). Latent profile analysis and logistic regression were performed.

**Results:**

The mean total score of discharge readiness among 356 elderly OVCF patients was 87.53 ± 16.90. Three latent profiles emerged as the optimal model: “Moderate Discharge Readiness Group” (15.2%), “High Discharge Readiness-Low Personal Status Group” (76.1%), and “Low Discharge Readiness-Low Adaptability Group” (8.7%). Multivariate logistic regression analysis revealed that comorbidities, length of stay in hospital, whether first hospitalization, and QDTS scores were factors influencing the potential discharge readiness profiles among elderly OVCF patients.

**Conclusion:**

Discharge readiness among elderly OVCF patients exhibits significant heterogeneity and is influenced by multifactorial, multilevel factors. Clinicians should not view these factors in isolation but rather understand their dynamic interplay across patient groups with varying readiness levels to implement the most targeted intervention strategies.

## 1 Introduction

With the accelerating global aging population, osteoporosis has emerged as a major chronic disease threatening elderly health. The incidence of one of its most common and severe complications—osteoporotic vertebral compression fractures (OVCF)—continues to rise, imposing significant disease burden and economic pressure on patients' families and healthcare system (1). Epidemiological data show that among people aged 40 and older in China, the prevalence of osteoporosis is 5.0% for men and 20.6% for women, while the prevalence of vertebral fractures is 10.5% and 9.7%, respectively (2). It is projected that by 2035, the annual incidence of osteoporosis-related fractures in China will reach 4.83 million cases, with annual healthcare expenditures estimated at approximately $19.92 billion (3).

Clinically, OVCF often causes chest or back pain, spinal deformity, and limited mobility, severely impacting patients' quality of life (4).

Currently, minimally invasive procedures such as percutaneous vertebroplasty (PVP) and percutaneous kyphoplasty (PKP) have become primary treatments for OVCF. These techniques effectively stabilize fractured vertebrae, alleviate pain, and facilitate early ambulation, thereby mitigating risks associated with prolonged bed rest (5–7). However, surgical interventions primarily address acute-phase issues, leaving post-discharge long-term management and rehabilitation as significant challenges. OVCF essentially represents an acute manifestation of chronic osteoporosis, requiring management to extend beyond hospital discharge into the home and community setting. This necessitates sustained adherence to standardized medication regimens, functional exercise programs, and lifestyle modifications. This patient population is typically elderly, often presenting with multiple comorbidities, and may experience cognitive decline and inadequate social support systems. These factors frequently leave patients ill-prepared for self-management after discharge.

Concurrently, the widespread adoption of Enhanced Recovery After Surgery (ERAS) protocols has significantly reduced the average length of stay in hospital for OVCF patients. While this trend improves the turnover efficiency of healthcare resources, it also necessitates that patients and caregivers acquire essential disease management knowledge and skills within a compressed timeframe. Consequently, it places higher demands on discharge readiness. Thus, scientifically assessing patients' preparedness for discharge and providing targeted interventions based on this assessment has become a critical challenge in orthopedic clinical nursing practice.

Discharge readiness refers to the level of preparedness patients possess across multiple dimensions—physical, psychological, and social—when transitioning from hospital to home or community settings (8). Adequate discharge planning can meet patients' practical needs after discharge by providing seamless, continuous care, thereby reducing readmissions, unplanned visits, and post-discharge difficulties, and ultimately improving patients' quality of life (8). Furthermore, effective discharge planning helps promote the rational use of healthcare resources and reduces overall healthcare costs (9). However, among elderly patients with OVCF, the following prominent issues exist: poor adherence to anti-osteoporosis medications and home rehabilitation exercises; and a decline in quality of life due to persistent pain and delayed functional recovery. It serves as a crucial indicator for assessing their ability to safely adapt to environmental changes. In recent years, research on discharge readiness has increased across various patient populations, yet studies focusing on the specific group of OVCF remain relatively limited. Existing studies often treat discharge readiness as a homogeneous concept, failing to reveal its potential heterogeneous manifestations across different dimensions (10).

Latent profile analysis (LPA), as an individual-centered statistical method, can identify distinct subgroups within a population based on patterns of responses to manifest indicators, thereby effectively revealing heterogeneous structures within the group (11). Compared to traditional data analysis methods, LPA focuses more on exploring group heterogeneity rather than overall relationships between variables, enabling more precise identification of diverse characteristics in patients' discharge readiness. Therefore, this study proposes to employ latent profile analysis to identify latent categories of discharge readiness among elderly OVCF patients. It further aims to analyze differences in general characteristics, clinical features, and health behaviors among patients in different categories, thereby providing evidence-based support for developing stratified, precision-targeted discharge guidance and intervention strategies.

## 2 Materials and methods

### 2.1 Design

This study is a cross-sectional study.

### 2.2 Participants and sample size

Convenience sampling was employed to recruit 356 elderly OVCF patients as study subjects between January and April 2025. All participants met the following inclusion criteria: (1)patients meeting the diagnostic criteria of the 2021 Expert Consensus on the Diagnosis and Treatment of Osteoporotic Vertebral Compression Fractures; (2) Age ≥ 60 years; (3) Being conscious and able to cooperate with the study; (4) Being volunteer to participate in this study. Exclusion criteria: (1) combination of other serious diseases (e.g., malignant tumor, serious cardiovascular and cerebrovascular diseases); (2) Having cognitive dysfunction or psychiatric diseases. Referencing similar studies, the sample size should reach 10–15 times the number of study variables (12). This study involved 22 variables, with a preliminary sample size requirement of 220–330 cases. Accounting for a 10% non-response rate, the minimum required sample size was 242 cases. The actual study enrolled 356 patients, meeting the sample size requirement.

### 2.3 Instruments

#### 2.3.1 General information questionnaire

Based on literature review and group discussions, researchers designed a general information questionnaire covering age, sex, ethnicity, marital status, place of residence, living conditions, education level, payment method of medical costs, Length of stay in hospital, residence status, Whether first hospitalization, reason for discharge, disease course, comorbidities, Whether surgery, and admission mode.

#### 2.3.2 Readiness for hospital discharge scale (RHDS)

The scale was originally developed by Wess et al. (13) and subsequently translated and adapted into Chinese by Lin et al. to form the Chinese version RHDS (14). This study utilized the Chinese version of the scale. It comprises three dimensions: Personal Status (3 items), Adaptive Capacity (5 items), and Anticipatory Support (4 items), totaling 12 items. Each item is scored on a scale of 0 to 10, with a maximum total score of 120. Higher scores indicate greater readiness for discharge. In this study, the Cronbach's α coefficient for this scale was 0.877.

#### 2.3.3 Quality of Discharge Teaching Scale (QDTS)

This scale was developed by Wess et al. (15). The Chinese version used in this study (16) comprises 24 items across three dimensions: Needed Content (6 items), actually Received Content (6 items), and Nurse Instruction Content and Skills (12 items), evaluating the quality of discharge instructions. Each item is scored on a scale of 0 to 10. A higher total scale score indicates a more favorable patient evaluation of discharge instruction quality. In this study, the Cronbach's α coefficient for this scale was 0.931.

### 2.4 Data collection

Researchers screened patients meeting inclusion criteria through medical record review. Data were collected via face-to-face interviews conducted on the day of patient discharge. Prior to questionnaire distribution, researchers explained the study objectives, significance, and completion procedures to obtain informed consent. For patients unable to self-administer the questionnaire, researchers completed it on their behalf after item-by-item inquiry.

### 2.5 Statistical analysis

Statistical analysis was performed using SPSS 24.0 software [IBM Corporation (IBM Corp.), Armonk, New York, USA]. Quantitative data are expressed as mean ± standard deviation (SD), while qualitative data are presented as case numbers and percentages (%). Chi-square tests or one-way ANOVA were used to compare differences in the characteristics of elderly OVCF patients across different discharge readiness profiles, firth regression were used for multivariate analysis, with the three potential categories of elderly OVCF patients as the dependent variables and the statistically significant factors from the univariate analysis as the independent variables. The “low discharge readiness–low adaptive capacity group” was used as the reference group, and the odds ratio (OR) and its 95% confidence interval (CI) were calculated for each independent variable. Common method bias was tested by unmeasured latent method construct (ULMC). Latent profile analysis was performed using Mplus 8.3 software (Muthén & Muthén, Los Angeles, California, USA) to identify distinct profiles of discharge readiness among elderly OVCF patients. Model fit was assessed using the following indices: (1) Information criteria: Akaike Information Criterion (AIC), Bayesian Information Criterion (BIC), and sample-corrected Bayesian Information Criterion (aBIC), where lower values indicate better model fit (17); (2)Entropy index: Reflecting classification accuracy, Entropy ≥ 0.800 indicates classification accuracy > 90 % (18); (3) Likelihood Ratio Test: Includes the Log-Likelihood Ratio Test (LMRT) and Bootstrap-based Likelihood Ratio Test (BLRT). A *P*-value < 0.05 indicates the k-profile model is significantly superior to the k-1-profile model (17). This study sets the significance level α = 0.05, meaning differences are considered statistically significant whenP < 0.05.

### 2.6 Ethical Considerations

This study was reviewed and approved by the Ethics Committee of Chengdu Third People's Hospital (Ethics Approval No.: 2024-S-384). All research procedures adhered to ethical guidelines.

## 3 Results

### 3.1 Common method bias assessment

Since both core variables were collected via patient self-reports, common method bias may be present. To conduct a rigorous analysis, we employed item parceling combined with a method of unmeasured latent method construct (19). First, we calculated the mean scores for each of the three dimensions of the RHDS and the three dimensions of the QDTS, yielding six observed indicators. We then constructed two latent variable models: Model 1 included only trait factors, while Model 2 added a common method factor to Model 1, assuming that the method factor was uncorrelated with the trait factors. We performed confirmatory factor analysis using maximum likelihood estimation. The results showed that the trait factor model fit well (CFI = 0.938, RMSEA = 0.126); after adding the method factor, model fit improved (ΔCFI = 0.062), but the average variance extracted (AVE) for the method factor was only 0.285, below the threshold of 0.30 (indicating that the method factor explained limited shared variance). Furthermore, Harman's single-factor test indicated that the first unrotated principal component explained 34.6% of the total variance, which is below the critical threshold of 40% (20). In summary, this study does not exhibit serious common-method bias.

### 3.2 Current status of discharge readiness among elderly OVCF patients

Among 356 elderly patients with OVCF, 141 were male (39.61%) and 215 were female (60.39%); ages ranged from 60 to 98 years, with a mean age of (72.90 ± 8.36) years; The total discharge readiness scores ranged from 32 to 112 points, with an average of 87.53 ± 16.90 points. Among the dimension scores, the Expected Support dimension scored highest (35.05 ± 5.21, item mean 8.77 ± 1.29), followed by the adaptive capacity dimension (35.49 ± 11.85, item mean 7.09 ± 2.37), while the personal status dimension scored relatively low (16.99 ± 3.75, item mean 5.66 ± 1.25). Detailed item scores are presented in Table 1.

**Table 1:** Scores on the discharge readiness scale for elderly OVCF patients.

Items	Full scores	Actual scores	Item mean scores
Personal status	30	16.99 ± 3.75	5.66 ± 1.25
1. Feeling unwell or in pain?	10		2.86 ± 1.84
2. How do you feel about your energy?	10		6.97 ± 2.03
3. How energetic you feel?	10		7.16 ± 2.20
Adaptive capacity	50	35.49 ± 11.85	7.09 ± 2.37
4. Ability to take care of your body when discharged from hospital	10		6.85 ± 2.77
5. care of your body when discharged from hospital, 5- Knowledge of the need to take care of oneself after being discharged from the hospital and returning home	10		7.43 ± 2.33
6. Ability to manage daily life at home,	10		6.79 ± 2.69
7. Ability to take care of oneself at home	10		6.80 ± 2.70
8. Degree to which you can perform medical care at home	10		7.62 ± 2.43
Expected Support	40	35.05 ± 5.21	8.77 ± 1.29
9. The extent of emotional support available after discharge	10		8.93 ± 1.39
10. The amount of assistance available for personal care after discharge	10		8.79 ± 1.43
11. The amount of assistance available for activities at home after discharge	10		8.49 ± 2.08
12. The amount of assistance available for medical care needs after discharge	10		8.86 ± 1.35
Total Scores	120	87.53 ± 16.90	7.30 ± 1.41

### 3.3 Latent profile analysis of discharge readiness in elderly OVCF patients

**Figure 1:**
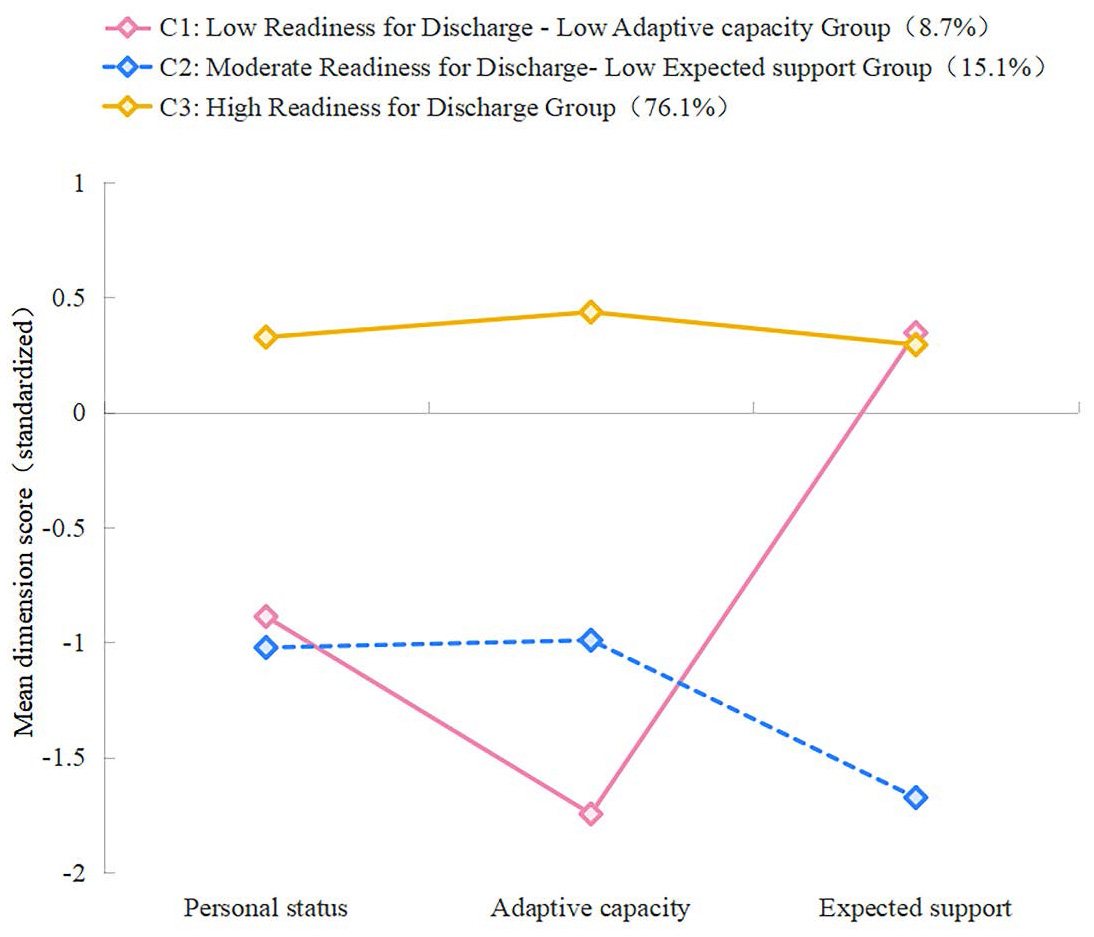
Latent types of discharge readiness in elderly OVCF patients. Scores for each dimension have been standardized to Z-scores to eliminate the impact of differences in the scoring ranges.

This study sequentially constructed latent profile models ranging from 1 to 4 based on the three dimensions of the Readiness for Discharge Scale as manifest indicators. Table 2 presents the results of the latent profile models. Findings indicate that AIC, BIC, and aBIC values decrease with increasing number of profiles. Model 4 failed to achieve statistical significance in the LMRT test (*P* = 0.2503) and was thus excluded. All models exhibited Entropy values >0.800, indicating 90% classification accuracy for the three latent profile models. Model 3 demonstrated the highest Entropy at 0.877, making it the optimal latent profile model. Figure 1 illustrates the scores across each dimension of discharge readiness for elderly OVCF patients across the three latent profiles. Based on the characteristics of the three potential subgroups, they were designated as the moderate discharge readiness–low expected support group (*n* = 54, 15.2%), the high discharge readiness group (*n* = 271, 76.1%), and the low discharge readiness–low adaptive capacity group (*n* = 31, 8.7%).

**Table 2:** Latent profile model of discharge readiness in elderly OVCF patients.

Model	AIC	BIC	aBIC	Entropy	P		Category
					**LMRT**	**BLRT**	
1	3039.848	3036.098	3044.063	–	–	–	–
2	2823.571	2862.320	2830.595	0.831	< 0.001	< 0.001	89/267
3	2772.091	2826.341	2781.926	0.877	0.0353	< 0.001	31/54/271
4	2737.907	2807.656	2750.552	0.859	0.2503	< 0.001	19/32/61/244

### 3.4 Univariate analysis of latent profiles of discharge readiness in elderly OVCF patients

Table 3 presents the results of univariate analysis for the three latent profiles of discharge readiness among elderly OVCF patients. Admission method (χ^2^ = 9.688, *P* = 0.008), length of stay in hospital (χ^2^ = 10.721, *P* = 0.03), disease course (χ^2^ = 10.348, *P* = 0.006), and whether first hospitalization (χ^2^ = 12.142, *P* = 0.002), comorbidities (χ^2^ = 14.489, *P* = 0.001), and QDTS (*F* = 15.201, *P* < 0.001) significantly influenced the latent profiles of discharge readiness among elderly OVCF patients.

**Table 3:** Univariate analysis of latent profiles of discharge readiness among elderly OVCF patients.

Variables		C1 (*N* = 54)	C2 (*N* = 271)	C3 (*N* = 31)	χ^2^/*F*	*P*
Sex	Male	14 (25.9%)	114 (42.1%)	13 (41.9%)	4.981	0.083
	Female	40 (74.1%)	157 (57.9%)	18 (58.1%)		
Age (year)	60–69	15 (27.8%)	120 (44.3%)	9 (29%)	8.250	0.083
	70–79	23 (42.6%)	98 (36.2%)	12 (38.7%)		
	≥80	16 (29.6%)	53 (19.6%)	10 (32.3%)		
Ethnicity	Han	52 (96.3%)	239 (88.2%)	30 (96.8%)	5.008	0.082
	Others	2 (3.7%)	32 (11.8%)	1 (3.2%)		
Education level	Primary school or below	40 (74.1%)	188 (69.4%)	23 (74.2%)	4.192	0.651
	Junior high school	10 (18.5%)	42 (15.5%)	5 (16.1%)		
	High school	3 (5.6%)	21 (7.7%)	2 (6.5%)		
	University or above	1 (1.9%)	20 (7.4%)	1 (3.2%)		
Marital status	Unmarried/divorced/widowed	21 (38.9%)	64 (23.6%)	8 (25.8%)	5.444	0.066
	Married	33 (61.1%)	207 (76.4%)	23 (74.2%)		
Payment method of medical costs	Medical insurance	51 (94.4%)	257 (94.8%)	28 (90.3%)	0.909	0.635
	Self-financed	3 (5.6%)	14 (5.2%)	3 (9.7%)		
Family per capita monthly income/Yuan	< 2,000	14 (25.9%)	53 (19.6%)	10 (32.3%)	7.022	0.135
	2,000–5,000	29 (53.7%)	148 (54.6%)	10 (32.3%)		
	>5,000	11 (20.4%)	70 (25.8%)	11 (35.5%)		
Geographic area of housing	Urban	32 (59.3%)	159 (58.7%)	15 (48.4%)	1.258	0.533
	Rural	22 (40.7%)	112 (41.3%)	16 (51.6%)		
Residence status	Living alone	11 (20.4%)	49 (18.1%)	3 (9.7%)	1.661	0.436
	Not living alone	43 (79.6%)	222 (81.9%)	28 (90.3%)		
Admission mode	Outpatient	36 (66.7%)	228 (84.1%)	23 (74.2%)	9.688	0.008
	Emergency	18 (33.3%)	43 (15.9%)	8 (25.8%)		
Whether surgery	yes	52 (96.3%)	240 (88.6%)	28 (90.3%)	2.971	0.226
	no	2 (3.7%)	31 (11.4%)	3 (9.7%)		
Length of stay in hospital/day	< 7	16 (29.6%)	75 (27.7%)	5 (16.1%)	10.721	0.03
	7–13	23 (42.6%)	145 (53.5%)	13 (41.9%)		
≥14	15 (27.8)	51 (18.8%)	13 (41.9%)		
Disease course/day	≤ 7	38 (70.4%)	237 (87.5%)	27 (87.1%)	10.348	0.006
	>7	16 (29.6%)	34 (12.5%)	4 (12.9%)		
Whether first hospitalization	yes	24 (44.4%)	159 (58.7%)	9 (29%)	12.142	0.002
	no	30 (55.6%)	112 (41.3%)	22 (71%)		
Reason for discharge	Physician advice	50 (92.6%)	246 (90.8%)	25 (80.6%)	3.642	0.162
	Voluntary request	4 (7.4%)	25 (9.2%)	6 (19.4%)		
Comorbidities	Yes	34 (63%)	96 (35.4%)	14 (45.2%)	14.489	0.001
	No	20 (37%)	175 (64.6%)	17 (54.8%)		
QDTS		149.02 ± 27.97	169.54 ± 24.82	166.48 ± 20.30	15.201	< 0.001

### 3.5 Multivariate analysis of latent profiles of discharge readiness in elderly OVCF patients

Due to the small sample sizes in some subgroups (the smallest subgroup had only 31 cases), there may be concerns regarding small-sample bias or complete separation. To address this, we used Firth-penalized likelihood logistic regression for robust estimation, using Group C1 as the reference group to compare C1 vs. C2 and C1 vs. C3, respectively. The results are shown in Table 4. In the C1 vs. C2 comparison, comorbidities and QDTS were independent risk factors; in the C2 vs. C3 comparison, admission mode, whether first hospitalizations, and length of stay were independent risk factors.

**Table 4:** Multivariate analysis of latent profiles of discharge readiness in elderly OVCF patients.

Group	Variables	B	SE	*P*	OR	95%CI
C1 VS. C2	Disease course	2.915	1.125	0.010	18.448	2.176–240.000
	Comorbidities	−1.211	0.527	0.022	0.298	(0.098, 0.828)
	Whether first hospitalization	−0.573	0.520	0.270	0.564	(0.193, 1.572)
	Admission mode	−1.661	0.972	0.088	0.190	(0.020,1.221)
	Length of stay in hospital (< 7 vs. ≥14)	0.429	0.742	0.563	1.535	(0.342, 7.089)
	Length of stay in hospital (7–13 vs. ≥14)	−0.051	0.565	0.928	0.950	(0.300, 2.926)
	QDTS	−0.033	0.012	0.005	0.967	(0.943, 0.989)
C1 VS. C3	Comorbidities	0.230	0.388	0.553	1.259	(0.571, 2.743)
	Whether first hospitalization	−1.054	0.403	0.009	0.348	(0.148, 0.767)
	Admission mode	−1.425	0.635	0.025	0.240	(0.070, 0.916)
	QDTS	0.007	0.008	0.369	1.007	(0.990, 1.024)
	Disease course	1.038	0.782	0.184	2.823	(0.584, 13.980)
	Length of stay in hospital (< 7 vs. ≥14)	1.333	0.547	0.015	3.792	(1.325, 12.350)
	Length of stay in hospital (7–13 vs. ≥14)	1.034	0.421	0.014	2.811	(1.208, 6.581)

*P*-values are based on the penalized likelihood ratio test; confidence intervals are based on the profile likelihood. Reference group: Low discharge readiness—low adaptive capacity group. In the comparison between C1 and C2, the confidence interval for disease course is extremely wide; this may be due to the small sample size or data outliers, so this result should be interpreted with caution.

## 4 Discussion

To our knowledge, this is the first study to explore latent categories of discharge readiness among elderly OVCF patients, yielding three key findings. First, we examined the current status of discharge readiness among elderly OVCF patients. Second, we identified three latent categories of discharge readiness among this population. Finally, we determined the factors influencing these latent categories of discharge readiness.

### 4.1 Current status of discharge readiness among elderly OVCF patients

The study results indicate that the total discharge readiness score for elderly OVCF patients was (87.53 ± 16.90), reflecting an overall moderately high level. This aligns with conclusions from similar studies, suggesting there remains room for improvement in discharge readiness for this population (21). The “Personal Status” dimension scored lowest, with most patients still experiencing pain at discharge and exhibiting insufficient energy and physical strength (corresponding to items 2 and 3). This reflects that symptom burden and inadequate physiological recovery are primary barriers to discharge confidence (22). First, persistent mechanical pain during movement or changes in body position directly reduces patients' physical comfort. Second, the restricted mobility caused by lumbar braces or the fear of falling limits daily activities, leading to a sense of helplessness. Third, this population commonly experiences a fear of re-fracture. Healthcare providers should emphasize individualized pain management prior to discharge, instruct patients on how to gradually increase activity while wearing a brace, and advise them on modifying their home environment to reduce the fear of falling. Regarding self-care (items 6 and 7), patients demonstrated low confidence, and they also harbored doubts about their ability to manage medical affairs (item 8), indicating insufficient capacity for independent health management post-discharge. Notably, the “Expected support” dimension scored highest, with particularly positive perceptions regarding emotional support (Item 9) and medical care assistance (Item 12). This indicates that family and social support systems serve as crucial protective resources during the discharge transition, consistent with Nurhayati et al.'s findings that robust social support enhances discharge readiness and improves health outcomes and clinical outcomes (23).International research also emphasizes that functional recovery and self-efficacy in orthopedic postoperative patients are key predictors of discharge readiness, exerting greater influence than knowledge-based education alone (24). Therefore, clinical nursing should prioritize effective interventions that help patients translate “knowing” into “doing.”

### 4.2 Latent categories of discharge readiness in elderly OVCF patients

This study employed latent profile analysis to categorize discharge readiness among elderly OVCF patients into three latent groups: low readiness for discharge-low adaptive capacity group, moderate readiness for discharge-low expected support group, and high readiness for discharge group. Among these, the group with high readiness accounted for the largest proportion, suggesting that most elderly OVCF patients can achieve a relatively high level of physical, psychological, and social readiness through standardized diagnosis, treatment, and care during hospitalization. A prominent characteristic of the group with moderate readiness is low expectations regarding the social or family support available after discharge. Although their own level of readiness is acceptable, they subjectively perceive a lack of support, which may lead to increased difficulties in coping after discharge. Clinical interventions for this group should prioritize strengthening the coordination of post-discharge support systems, such as scheduling follow-up phone calls, home visits, or community rehabilitation guidance to help establish an accessible support network. The low readiness group scored lowest on the adaptive capacity dimension, indicating insufficient preparation for post-discharge life. Due to inadequate self-adaptation abilities, this group requires higher levels of family and professional care support, placing them at high risk for adverse outcomes (e.g., readmission, delayed recovery) after discharge. For such patients, establishing a coordinated “hospital-community-home” continuity of care pathway is recommended: Pre-discharge rehabilitation therapists should provide intensive instruction on basic living skills like assistive device usage. At discharge, seamless coordination with community health centers should be initiated to pre-schedule follow-up visits and home-based rehabilitation guidance, thereby mitigating rehabilitation risks stemming from inadequate adaptive capacity.

### 4.3 Analysis of factors influencing potential discharge readiness profiles among elderly OVCF patients

This study found that elderly OVCF patients with comorbidities were more likely to be classified into the low discharge readiness group, consistent with the findings of Yuan Jing et al. regarding postoperative cervical cancer patients (25). The coexistence of multiple chronic diseases exacerbates patients' physical functional impairment, delays the rehabilitation process, reduces their confidence in self-management after returning home, and is accompanied by significant psychological distress (26).Furthermore, long-term medication and treatment burdens increase patients' cognitive load, while multiple coexisting conditions often bring anxiety and depression, further diminishing their psychological resilience to cope with new fractures. Therefore, healthcare providers should prioritize these patients during health education. Continuous support can be enhanced by distributing medication guidance booklets at discharge and providing timely medication guidance through “Internet+” platforms.

Regarding length of stay in hospital, both “ < 7 days” (OR = 3.792) and “7–13 days” (OR = 2.811) yielded OR values greater than 1 with significant *P*-values. This indicates that patients with shorter or moderate hospital stays were more likely to enter the high preparedness group compared to those hospitalized for ≥14 days. Typically, prolonged hospitalization often signifies more complex conditions, increased complications, or slower recovery. These factors impose sustained burdens on patients' physical, psychological, and social support systems, thereby weakening their readiness for discharge. Conversely, meeting discharge criteria within a shorter timeframe frequently indicates favorable physical condition and rehabilitation potential, which helps bolster confidence in discharge readiness. This finding contrasts with a study on ischemic stroke patients (27), which suggested longer hospital stays may correlate with higher readiness levels. This discrepancy likely stems from differing rehabilitation pathways: stroke patients require more systematic in-hospital rehabilitation training, and extended stays may indicate more thorough functional assessments and guidance.

Additionally, whether first hospitalizations (OR = 0.348, *P* = 0.009) suggests that patients admitted for the first time tend to be better prepared; this finding is consistent with the conclusions of a study on patients following craniotomy, which reported that first-time inpatients were better prepared for discharge (28). Possible reasons include the fact that first-time inpatients often lack prior experience with disease management and are more receptive to and reliant on discharge instructions, leading them to take the discharge preparation process more seriously. Furthermore, multiple hospitalizations may themselves reflect more complex medical conditions, a higher number of comorbidities, or poorer self-management skills; these factors collectively contribute to reduced readiness for discharge.

Previous studies have shown that the QDTS is a significant predictor of discharge readiness (29–31). In this study, the OR value for QDTS in the “moderate readiness vs. low readiness” comparison was 0.967, meaning that for every 1-point increase in the score, the likelihood of patients belonging to the moderate readiness group decreased by approximately 3.3%. This counterintuitive finding holds significant implications: For poorly prepared patients with weaker baseline conditions, detailed and high-standard discharge instructions may exceed their capacity to absorb information. Such overload may even heighten anxiety, paradoxically highlighting their inadequate preparedness (32). Therefore, for this group, discharge instructions should follow the principle of “less is more, with repetition of key points,” focusing on the most essential self-care skills (such as medication management, symptom recognition, and emergency response), and avoiding the one-time delivery of excessive information.

In this study, disease course showed a significant and extremely high odds ratio (OR = 18.45) only in the comparison between the low-readiness group (C1) and the moderate-readiness group (C2), while its confidence interval was very wide (2.18–240.00), spanning nearly two orders of magnitude. Such extreme estimates are typical of small samples or cases of complete separation: it is possible that, in the sample of this study, the variable “duration of illness” was almost completely separated between groups C1 and C2. Although Firth-penalized likelihood regression converges and provides finite estimates, it still produces extremely large and unstable OR values in cases of severe separation. Therefore, we cautiously recommend against placing excessive clinical weight on the significant results regarding duration of illness. Future studies should validate the true association between disease duration and readiness for discharge in larger samples and consider using Bayesian methods or regularized regression to further stabilize the estimates.

## 5 Limitations

This study has certain limitations. First, participants were drawn exclusively from a single hospital, limiting sample representativeness and restricting the generalizability of findings to broader populations of elderly OVCF patients. Second, the small sample size resulted in uneven distribution across groups, particularly the low discharge readiness group (*n* = 31), potentially affecting the stability and reliability of statistical results. Additionally, while the study incorporated multiple common clinical variables, it did not include important psychosocial factors such as health literacy and psychological resilience, which may also significantly influence discharge readiness. Future research will aim to validate and expand these findings by increasing sample size, conducting multicenter collaborations, and integrating objective measures with qualitative data.

## 6 Conclusion

This study investigated discharge readiness among 356 elderly OVCF patients, employing latent profile analysis to identify intrinsic heterogeneity and explore associated factors. Results revealed substantial variation in discharge readiness among elderly OVCF patients, which could be categorized into three distinct latent classes: “low,” “moderate,” and “high.” Admission mode, comorbidities, QDTS, whether first hospitalization, and length of stay in hospital emerged as independent factors distinguishing these categories.

## Data Availability

The original contributions presented in the study are included in the article/supplementary material, further inquiries can be directed to the corresponding author.
